# Virtual Reality Goggles as a Distraction Technique for Managing Behavioral Conduct During Pulp Pediatric Dental Treatment: A Systematic Review and Meta-Analysis

**DOI:** 10.3390/dj14080517

**Published:** 2026-08-13

**Authors:** Carmen Machuca-Portillo, Maria Biedma-Perea, Cira Suárez-Marchena, Carolina Caleza-Jiménez, Lucy Chandler-Gutiérrez, Lydia López-del Valle, Juan J. Segura-Egea

**Affiliations:** 1 Department of Stomatology, Pediatric Dentistry Division, School of Dentistry, University of Sevilla, C/Avicena s/n, 41009 Sevilla, Spain; mmachuca@us.es (C.M.-P.); mbiedma1@us.es (M.B.-P.); chandler@us.es (L.C.-G.); 2 School of Dental Medicine, University of Puerto Rico, Medical Sciences Campus UPR, Building San Juan Medical Center, San Juan, PR 00936, USA; lydia.lopez1@upr.edu; 3 Department of Stomatology, Endodontic Division, School of Dentistry, University of Sevilla, C/Avicena s/n, 41009 Sevilla, Spain; segurajj@us.es

**Keywords:** virtual reality, pediatric dentistry, pulp therapy, dental anxiety, pain perception, behavior management

## Abstract

**Objective:** To systematically evaluate the effectiveness of virtual reality (VR) distraction systems in reducing pain perception and dental anxiety during pediatric pulp therapy procedures. **Methods:** A systematic review and meta-analysis was conducted according to PRISMA 2020 guidelines and registered in PROSPERO (CRD42026139774). PubMed/MEDLINE, Scopus, and Embase were searched for randomized clinical trials evaluating immersive VR distraction during pediatric pulp therapy. Risk of bias was assessed using RoB 2, and certainty of evidence was evaluated with GRADE. Random-effects meta-analysis was performed for pain outcomes. **Results:** Seven randomized clinical trials were included in the qualitative synthesis. Three parallel-group randomized controlled trials were included in the pain meta-analysis. VR distraction significantly reduced self-reported pain (SMD) = −1.28 (95% C.I. = −2.49 to −0.07; *p* = 0.038) compared with control interventions. Substantial heterogeneity was observed for both outcomes (I^2^ > 93%). Most studies also reported improvements in behavioral cooperation, whereas physiological measures showed inconsistent findings. The certainty of evidence was rated as low for pain and anxiety outcomes. **Conclusions:** VR distraction may represent a promising non-pharmacological behavioral management strategy for reducing pain perception and dental anxiety during pediatric pulp therapy. However, these findings should be interpreted with caution because they are based on a small number of studies with substantial methodological heterogeneity. Further well-designed randomized clinical trials using standardized intervention protocols and outcome measures are required before firm conclusions or recommendations for routine clinical implementation can be established.

## 1. Introduction

Oral health is an essential component of child well-being and represents a determining factor in quality of life among children. However, fear and anxiety associated with dental treatment remain among the main barriers to pediatric dental care. Various studies have reported that the prevalence of dental fear and anxiety ranges between 20% and 45%, depending on age and sociocultural context [1,2].

Pediatric pulp therapy procedures are among the most invasive treatments commonly performed in primary dentition and may provoke pain, fear, and anxiety. Although these concepts are closely related, they represent distinct clinical outcomes. Pain refers to the unpleasant sensory and emotional experience associated with tissue injury, whereas dental anxiety reflects anticipatory emotional distress related to dental treatment. Both factors may trigger emotional and physiological responses, including crying, movement, treatment interruption, increased heart rate, and elevated cortisol levels, which may adversely affect children’s behavior and cooperation during treatment and compromise the quality and efficiency of care [3,4,5]. Likewise, fear and anxiety may lead to maladaptive behaviors and poor cooperation during dental procedures, increasing the likelihood of postponing, interrupting, or avoiding dental care [6,7]. Consequently, effective behavioral management strategies should aim not only to reduce pain and anxiety but also to improve treatment acceptance, cooperation, and overall patient experience.

In pediatric dentistry, behavioral management plays a key role in ensuring effective treatment and positive patient experiences. Traditionally, dentists have relied on multiple behavior management techniques, including tell-show-do, positive reinforcement, behavior modeling, voice control, contingency management, escape control, physical restraints, sedation, and distraction [8]. However, some of these strategies have generated controversy because of their potential to negatively affect children’s psychological well-being and restrict their autonomy [9]. Consequently, there is growing interest in implementing less aversive and more effective approaches that facilitate treatment while reducing pain and anxiety.

Among contemporary behavioral management strategies, distraction techniques have gained increasing relevance as methods for modulating pain perception and reducing anxiety during invasive procedures [10,11,12,13]. Distraction is defined as a mental state in which attention is shifted from an unpleasant stimulus to a more pleasant one, thereby reducing the perception of the harmful stimulus through reality alteration [14]. According to the American Academy of Pediatric Dentistry [15], distraction is indicated for pediatric patients of any age and is considered an effective technique for reducing pain-related responses during dental procedures.

Among the available distraction modalities, audiovisual media, television, virtual reality (VR), and audiovisual headsets have demonstrated usefulness in diverting children’s attention and facilitating treatment [10]. In recent years, VR and other immersive technologies have attracted increasing interest in healthcare research [16]. Although initially developed for recreational purposes, these technologies have progressively expanded into clinical settings, particularly for the management of pain and treatment-related distress [11,17]. VR enables users to immerse themselves in virtual environments capable of partially isolating them from external stimuli through audiovisual devices that integrate images and sound in real time. Hoffman et al. [18] described VR headsets as systems that project images directly onto the user’s eyes while blocking environmental stimuli, thereby promoting a greater sense of immersion. To maximize effectiveness, the presented content should capture and maintain the child’s attention [19]. In this regard, cartoons and other audiovisual content have demonstrated considerable value as distraction tools because they are easy to administer and highly engaging.

The relevance of these strategies is particularly evident during pediatric pulp treatments, which require adequate pain and anxiety management to ensure patient comfort, cooperation, and adherence to treatment [20,21].

Despite the increasing use of virtual reality technologies in pediatric dentistry, the available evidence remains fragmented. Previous studies have evaluated VR distraction across a broad range of dental procedures, including preventive, restorative, and surgical treatments, with limited attention specifically directed toward pulp therapy procedures [22]. In addition, considerable variability exists regarding the types of VR devices employed, the level of immersion provided, and the instruments used to assess pain, anxiety, behavioral responses, and physiological outcomes [22]. This methodological heterogeneity complicates the interpretation of findings and limits the ability to draw procedure-specific conclusions. Furthermore, to our knowledge, no previous systematic review and meta-analysis has specifically evaluated the effectiveness of immersive VR distraction during pediatric pulp therapy procedures. Therefore, the present study aimed to systematically review and quantitatively synthesize the available evidence regarding the effects of VR distraction on pain perception, dental anxiety, and behavioral outcomes in children undergoing pulp therapy.

## 2. Materials and Methods

### 2.1. Protocol and Registration

This systematic review was designed and reported in accordance with the Preferred Reporting Items for Systematic Reviews and Meta-Analyses (PRISMA) 2020 statement [23] (Table S1). The study protocol was prospectively registered in the International Prospective Register of Systematic Reviews (PROSPERO; CRD42026139774, accessed on 21 May 2026).

### 2.2. Review Question

The review question was developed according to the PICO (Population, Intervention, Comparison, Outcome) framework, as described by Hoogendam et al. [24].

Population (P): children ≤ 12 years of age undergoing pulp therapy dental procedures.

Intervention (I): use of immersive virtual reality distraction systems.

Comparison (C): conventional behavior management techniques or standard care.

Outcome (O): Primary outcomes: reduction in pain perception and dental anxiety during pediatric pulp therapy procedures.

Secondary outcomes: behavioral response/cooperation and physiological stress-related parameters, including salivary cortisol levels, heart rate, and pulse rate.

Based on this framework, the following review question was formulated:

In children undergoing pulp therapy dental procedures, does immersive virtual reality distraction reduce pain and anxiety compared with conventional behavior management techniques?

### 2.3. Eligibility Criteria

Studies were considered eligible if they fulfilled all of the following criteria:

Study design: randomized controlled trials, split-mouth randomized clinical trials, or controlled clinical trials.

Population: healthy children aged ≤ 12 years undergoing pulp therapy procedures in primary teeth. The upper age limit of 12 years was selected because the review specifically focused on pulp therapy procedures performed in primary dentition. This age range encompasses the period during which primary teeth are generally retained and such procedures are most commonly indicated, thereby ensuring a clinically homogeneous study population.

Dental procedures: pulpotomy, pulpectomy, vital pulp therapy, or other pulp treatment procedures performed in primary dentition.

Intervention: immersive virtual reality systems using head-mounted displays or VR goggles designed to provide audiovisual distraction during treatment.

Comparator: conventional behavior management techniques, standard dental care, or no distraction intervention.

Primary outcomes: self-reported pain perception and/or dental anxiety assessed using validated instruments (e.g., Wong–Baker Faces Pain Rating Scale, Visual Analog Scale, Modified Child Dental Anxiety Scale, Facial Image Scale, or other validated pediatric instruments).

Secondary outcomes: behavioral response/cooperation and physiological stress-related parameters, including salivary cortisol levels, heart rate, and pulse rate.

Publication characteristics: full-text articles published in peer-reviewed journals in English between January 2016 and May 2026.

Studies were excluded if they:

Included adults or participants older than 12 years.

Included children with systemic diseases, neurological disorders, developmental disorders, special healthcare needs, or conditions that could influence anxiety, pain perception, or behavioral responses. Children with developmental disorders, neurological conditions, cognitive impairments, or other special healthcare needs were excluded to reduce clinical heterogeneity and improve comparability among studies. This criterion was not intended to imply that VR interventions are ineffective in these populations; rather, the present review focused on generally healthy children undergoing pulp therapy procedures.

Evaluated dental procedures other than pulp therapy.

Used non-immersive distraction methods without a virtual reality component.

Did not report at least one predefined outcome of interest.

Were reviews, meta-analyses, case reports, case series, conference abstracts, editorials, letters, commentaries, or expert opinions.

Did not provide sufficient methodological information for data extraction.

### 2.4. Search Strategy

A comprehensive and systematic literature search was conducted in the PubMed/MEDLINE, Scopus, and Embase databases.

The search strategy combined controlled vocabulary terms (MeSH and Emtree) and free-text keywords related to pediatric dentistry, pulp therapy procedures, virtual reality distraction, dental anxiety, pain perception, and behavior management.

The following key concepts were included in the search strategy: pediatric patients, pulp therapy, pulpotomy, pulpectomy, dental anxiety, pain perception, virtual reality, immersive distraction, and audiovisual distraction.

Boolean operators (“AND”, “OR”) were used to combine search terms appropriately, and truncation operators were applied when relevant to improve search sensitivity.

The search strategy was adapted for each database according to its specific indexing system and search syntax. The final electronic search was conducted on 1 May 2026. No restrictions regarding study design were applied during the initial search process to maximize sensitivity. No language restrictions were applied during database searching. The publication date restriction (January 2016 to May 2026) was incorporated into the search strategy to focus on contemporary VR technologies, whereas language eligibility (English) was applied during the screening phase.

Filters for publication date (last 10 years) and language (English) were applied during the screening phase. The publication date restriction was applied because virtual reality technologies have undergone considerable advances in immersion, display quality, portability, and user interaction over the last decade. Earlier-generation systems differ considerably from contemporary VR devices and may not adequately reflect current clinical applications. Therefore, restricting the review to recent studies was considered appropriate to maximize the clinical relevance and applicability of the findings [25,26].

The complete electronic search strategies for each database are presented in Table 1 to ensure reproducibility.

### 2.5. Selection of Studies

Study selection was independently performed by three reviewers (C.M.-P., C.S.-M., and L.C.-G.) in two phases. First, titles and abstracts were screened to identify potentially eligible studies. Subsequently, full-text articles were assessed according to the predefined inclusion and exclusion criteria.

Any disagreements among reviewers were resolved through discussion until consensus was reached.

Additionally, the reference lists of all included studies were hand-searched to identify any additional relevant publications.

### 2.6. Data Extraction

Data extraction was independently performed by two reviewers (C.M.-P. and C.S.-M.) and subsequently verified by a third reviewer (J.J.S.-E.) to ensure data accuracy and consistency. Any disagreements were resolved by consensus.

The following variables were extracted from each included study: authors and year of publication, country of origin, study design, sample size and participants’ mean age, type of pulp therapy procedure, characteristics of the virtual reality intervention, control group characteristics, anxiety and pain assessment methods physiological outcome measures, and main findings and conclusions.

All extracted data were systematically tabulated. A standardized data extraction form was developed prior to data collection.

### 2.7. Data Analysis and Meta-Analysis

Data extracted from the included studies were summarized descriptively in evidence tables, including study characteristics, sample size, intervention details, assessment methods, and main outcomes related to pain perception and dental anxiety. Due to the variability in assessment instruments among studies, outcomes were grouped according to the construct evaluated.

A quantitative meta-analysis was performed when at least three parallel-group randomized controlled trials reported comparable continuous outcomes (mean, standard deviation, and sample size). In the present review, only self-reported pain fulfilled this criterion. Dental anxiety outcomes were synthesized narratively because, after exclusion of crossover and split-mouth trials from the quantitative synthesis, only two parallel-group randomized controlled trials remained, which was considered insufficient for a robust meta-analysis.

Although all studies included in the quantitative synthesis assessed pain using the Wong–Baker Faces Pain Rating Scale (WBFPS), standardized mean differences (SMDs) were retained because this approach had been prespecified in the review protocol and facilitates comparison with future meta-analyses that may include different validated pain scales. Dental anxiety outcomes assessed with the Modified Child Dental Anxiety Scale (MCDAS), Venham Clinical Anxiety Rating Scale (VCARS), Facial Image Scale (FIS), and other validated instruments were synthesized narratively.

Because different validated scales were used to assess similar outcomes, pooled effect estimates were calculated using standardized mean differences (SMDs) with 95% confidence intervals (CIs). A random-effects model was applied to account for expected methodological and clinical heterogeneity among studies, including differences in study design, participant characteristics, and dental procedures performed. Statistical heterogeneity was assessed using the I^2^ statistic, with values above 50% considered indicative of substantial heterogeneity.

Randomized crossover and split-mouth trials were considered eligible for the qualitative synthesis. However, these studies were not included in the quantitative synthesis because the original publications did not report the paired standard deviations, standard errors, or within-subject correlation coefficients required to appropriately account for the paired nature of the data, as recommended by the Cochrane Handbook for Systematic Reviews of Interventions (Cochrane Handbook for Systematic Reviews of Interventions. Version 6.5). To avoid violating the assumptions of the meta-analysis, only parallel-group randomized controlled trials were included in the quantitative synthesis.

Studies lacking sufficient quantitative data for statistical pooling were narratively synthesized. Outcomes related to physiological parameters, including salivary cortisol levels and pulse/heart rate measurements, were not included in the quantitative synthesis due to heterogeneity in measurement protocols and insufficient comparable data across studies.

To explore the robustness of the pooled estimates and the influence of individual studies, a leave-one-out sensitivity analysis was performed for the pain meta-analysis. Formal subgroup analyses and meta-regression were not conducted because of the small number of studies included in the quantitative synthesis, which would have provided insufficient statistical power and potentially unreliable estimates.

### 2.8. Risk of Bias Assessment

The methodological quality of the included randomized clinical trials was assessed using the revised Cochrane Risk of Bias tool for randomized trials (RoB 2) [27].

The following domains were evaluated: bias arising from the randomization process; bias due to deviations from intended interventions; bias due to missing outcome data; bias in outcome measurement; and bias in selection of the reported results.

Each domain was classified as “low risk of bias,” “some concerns,” or “high risk of bias,” according to the RoB 2 guidance.

Two independent reviewers (C.M.-P. and J.J.S.-E.) performed the risk of bias assessment independently. Any disagreements were resolved through discussion until consensus was reached.

### 2.9. Certainty of Evidence Assessment (GRADE)

The certainty of evidence for the main outcomes was assessed using the Grading of Recommendations Assessment, Development and Evaluation (GRADE) approach [28].

The following domains were evaluated: risk of bias, inconsistency, indirectness Imprecision, and publication bias (when assessable). The certainty of evidence was classified as high, moderate, low, or very low.

Three reviewers (C.M.-P., C.S.-M., and L.C.-G.) independently assessed each domain. Any disagreements were resolved through discussion until consensus was reached.

## 3. Results

### 3.1. Study Selection

**Figure 1:**
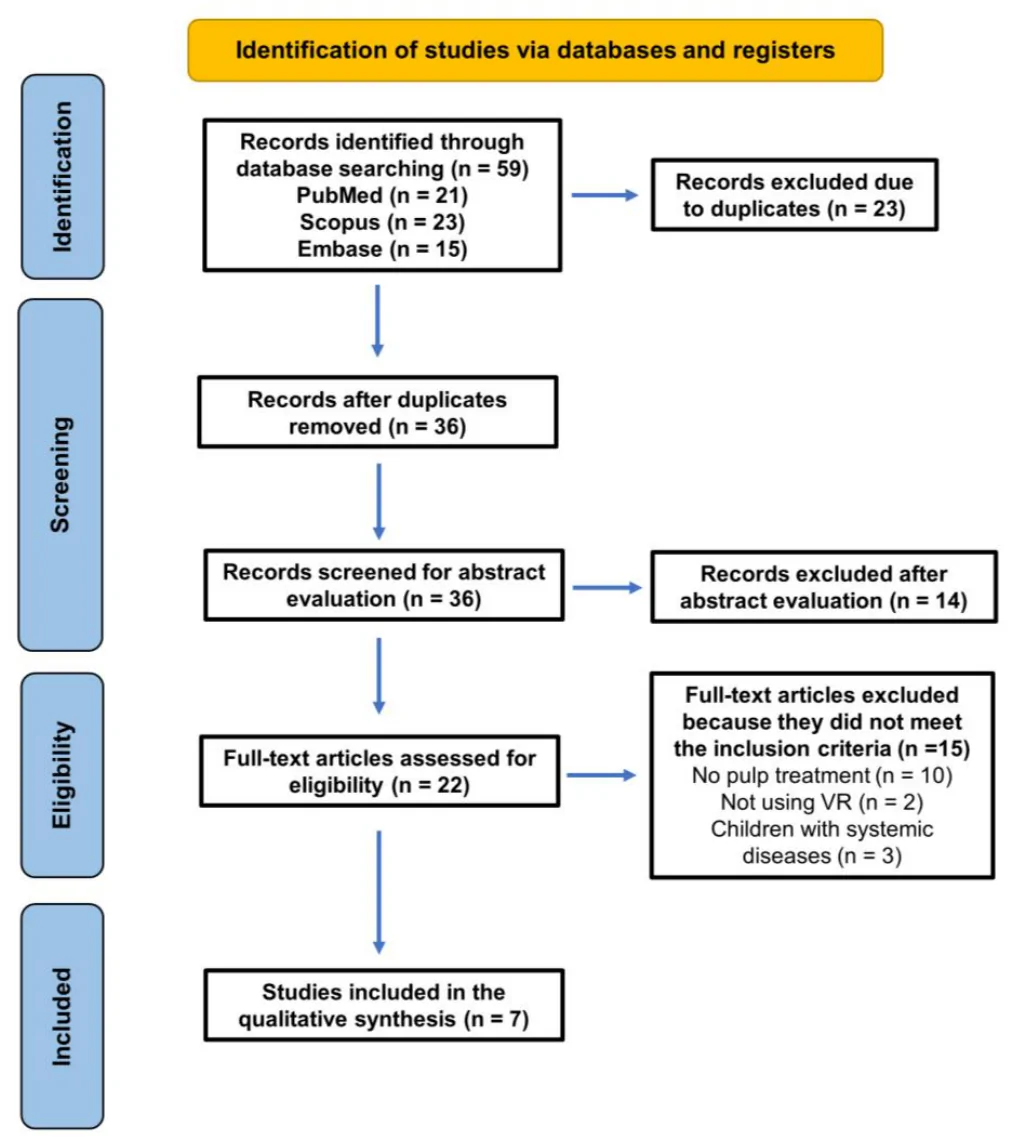
PRISMA flow diagram illustrating the study selection and screening process.

A total of 59 records were identified through database searching, including PubMed (*n* = 21), Scopus (*n* = 23), and Embase (*n* = 15) (Figure 1). After removing 23 duplicate records, 36 studies remained for abstract screening. Following abstract evaluation, 14 records were excluded. Subsequently, 22 full-text articles were assessed for eligibility, of which 15 were excluded for not meeting the inclusion criteria: not related to pulp treatment (*n* = 10), not using virtual reality (*n* = 2), and inclusion of children with systemic diseases (*n* = 3) (Table 2). Finally, seven studies were included in the qualitative synthesis.

### 3.2. Characteristics of the Included Studies

All included studies were randomized clinical trials, including parallel-group randomized controlled trials and randomized crossover designs [44,45,46,47,48,49,50]. No longitudinal or prospective cohort studies meeting inclusion criteria were identified. The characteristics of the seven included studies [44,45,46,47,48,49,50] are summarized in Table 3. The studies were published between 2018 [44] and 2025 [45,46] and mainly consisted of randomized clinical trials conducted in children undergoing different dental procedures. Sample sizes ranged from 20 [49] to 154 [45] participants, with children aged approximately 4 [44,48,49] to 12 years [45]. The studies investigated the effect of VR distraction on pain perception and anxiety response during dental procedures, particularly during pulp therapy treatments.

Various validated assessment tools were used to evaluate anxiety and pain outcomes. The most frequently used instrument was the Wong–Baker Faces Pain Rating Scale [44,45,47,48,50], which was applied in most included studies. Other assessment methods included the Modified Child Dental Anxiety Scale [44,47,50], Facial Image Scale (FIS) [46], Children’s Fear Survey Schedule–Dental Subscale (CFSS-DS) [46], Visual Analog Scale (VAS) [45], Face, Legs, Activity, Cry, Consolability Scale (FLACC) [45], Venham Clinical Anxiety Rating Scale (VCARS) [49], Venham Picture Scale (VPS) [48], salivary cortisol measurements [47,49], and pulse/heart rate monitoring [44,46,50].

### 3.3. Data Analysis: Qualitative Synthesis

Several studies demonstrated significant reductions in pain and anxiety among pediatric patients receiving VR distraction. Shetty et al. [47] reported a significant decrease in both pain perception and state anxiety (*p* < 0.001 and *p* = 0.002, respectively), accompanied by a marked reduction in salivary cortisol levels, supporting both subjective and physiological benefits. Similarly, Niharika et al. [44] observed significant improvements in pain perception and anxiety scores during dental treatment (*p* < 0.001). El Haleem et al. [48] also reported significant improvements in child behavior, pain perception, and anxiety scores (*p* < 0.05) with the use of VR eyeglasses during dental treatment. Likewise, Bahrololoomi et al. [50] found significantly lower Modified Child Dental Anxiety Scale scores (*p* = 0.02) and Wong–Baker Faces Pain Scale scores (*p* = 0.001) in the VR group, although pulse rate did not differ significantly between groups. Alshatrat et al. [45] further noted that VR was particularly effective during painful procedures involving local anesthesia administration, where improvements in subjective and behavioral outcomes were observed (*p* < 0.05). Kasimoglu et al. [46] observed that pulse rates significantly decreased in the VR group after treatment compared with pretreatment values (*p* < 0.05).

However, not all findings uniformly supported the effectiveness of VR across all outcome measures. Kasimoglu et al. [46] found no statistically significant differences between groups in terms of Facial Image Scale and Children’s Fear Survey Schedule–Dental Subscale scores before and after the procedure (*p* > 0.05). Mahmoud et al. [49] reported significantly lower Venham Clinical Anxiety Rating Scale scores in the VR group during intraoral examination (*p* = 0.02), indicating reduced clinical anxiety; nevertheless, no significant changes in salivary cortisol levels were observed.

### 3.4. Data Analysis: Quantitative Analysis and Meta-Analysis

A quantitative meta-analysis was performed to evaluate the effect of virtual reality distraction on self-reported pain during pediatric dental treatment. Dental anxiety outcomes were synthesized narratively because only two parallel-group randomized controlled trials remained after exclusion of crossover and split-mouth studies from the quantitative synthesis.

Three parallel-group randomized controlled trials were included in the meta-analysis evaluating self-reported pain [45,47,48]. Pain outcomes were assessed using the Wong–Baker Faces Pain Rating Scale (WBFPS), which was consistently reported across all included studies. A total of 209 children (105 in the VR groups and 104 in the control groups) were analyzed, including participants receiving VR distraction and control interventions without VR (Table 4).

Children treated with VR reported lower pain scores compared with control groups during invasive dental procedures, particularly during pulp therapy and procedures requiring local anesthesia administration. All three included studies reported lower pain scores in the VR group than in the control group.

**Figure 2:**
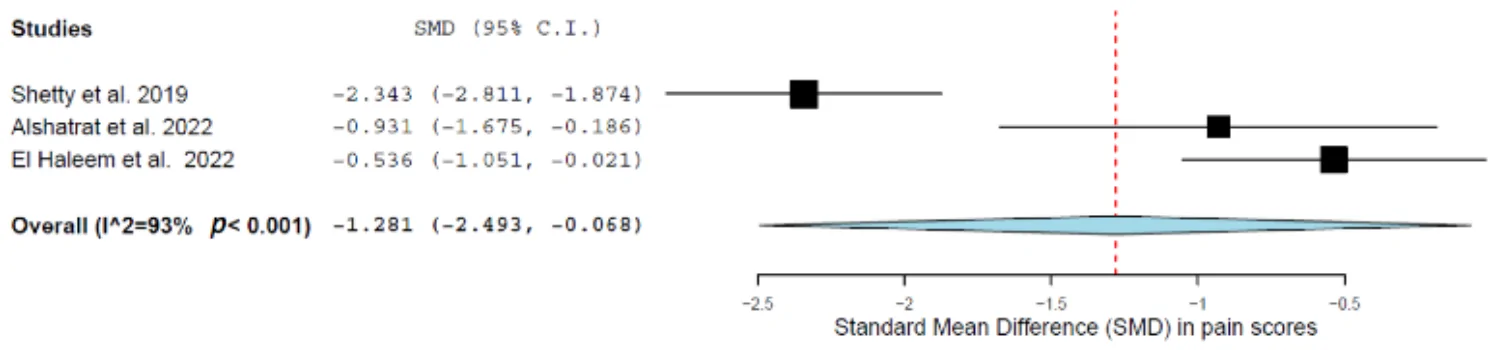
Forest plot of the meta-analysis evaluating the effect of virtual reality distraction on self-reported pain during pediatric dental treatment. Negative standardized mean difference (SMD) values favor the virtual reality intervention. The pooled random-effects model demonstrated a significant reduction in pain scores in the VR group compared with controls (SMD = −1.28; 95% C.I. = −2.49 to −0.07; *p* = 0.038).The vertical red dotted line represents the line of no effect (SMD = 0).Leave-one-out sensitivity analysis (Figure 3) showed that the direction of the pooled effect consistently favored VR distraction after sequential exclusion of each study, with SMD values ranging from −0.664 (95% CI: −1.087 to −0.240) to −1.667 (95% CI: −3.050 to −0.285). Although the magnitude of the effect varied because only three studies were available, no individual study completely changed the overall interpretation of the findings [45,47,48].

The pooled analysis using a random-effects model demonstrated a statistically significant reduction in self-reported pain in favor of the VR intervention (standardized mean difference [SMD] = −1.28 (95% C.I. = −2.49 to −0.07; *p* = 0.038) (Figure 2). This result suggests that VR significantly reduced self-reported pain during pediatric dental procedures compared with control interventions.

However, significant heterogeneity was observed among the included studies (I^2^ = 93%; Tau^2^ = 1.059; *p* < 0.001), suggesting considerable variability in study characteristics, intervention protocols, and pain assessment conditions. In practical terms, this indicates that the included studies differed substantially in their participant characteristics, VR interventions, clinical procedures, and outcome assessment methods. Consequently, although all studies consistently favored VR distraction, the exact magnitude of the pooled treatment effect should be interpreted with caution. The principal contribution of the present meta-analysis lies in the consistent direction of effect favouring VR distraction rather than in the precise magnitude of the pooled standardized mean difference.

**Figure 3:**
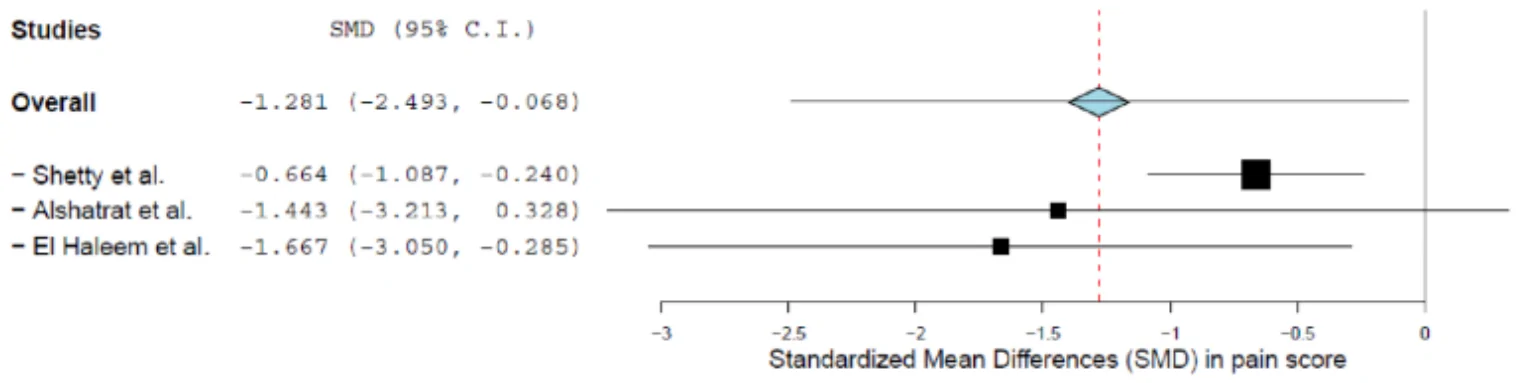
Leave-one-out sensitivity analysis for self-reported pain. The pooled standardized mean difference (SMD) remained consistently in favor of virtual reality distraction after sequential exclusion of each study, indicating that no single study substantially influenced the overall result. The vertical red dotted line represents the line of no effect (SMD = 0) [45,47,48].

### 3.5. Risk of Bias Assessment

The methodological quality of the randomized clinical trials was assessed using the Cochrane Risk of Bias 2 (RoB 2) tool [27] (Table 5). Most studies were judged as presenting “some concerns” overall, mainly due to insufficient information regarding allocation concealment, blinding procedures, and selective reporting. Only one study was considered at low risk of bias across all evaluated domains [45]. No study was classified as having a high risk of bias.

### 3.6. GRADE Assessment of the Certainty of Evidence

According to the GRADE approach, the certainty of evidence supporting the effectiveness of virtual reality distraction for reducing self-reported pain was rated as low. Although dental anxiety outcomes were synthesized narratively rather than quantitatively, the certainty of evidence supporting this outcome was also judged to be low because of methodological limitations, inconsistency across studies, and imprecision. The certainty of evidence for physiological outcomes was considered very low owing to the limited number of studies, inconsistency of findings, and considerable heterogeneity (Table 6).

The certainty of evidence for dental anxiety was assessed based on qualitative evidence because a quantitative synthesis was not performed after exclusion of crossover and split-mouth trials from the meta-analysis.

## 4. Discussion

In recent years, virtual reality (VR) has gained increasing attention in clinical research as an innovative distraction technique for pain and anxiety management. VR headsets may provide superior distraction compared with conventional audiovisual systems because they display immersive images directly in front of the user’s eyes while simultaneously blocking external visual and auditory stimuli from the real environment. As a result, the child’s attention is redirected away from the dental procedure, thereby reducing awareness of unpleasant stimuli and potentially improving behavioral cooperation during treatment.

The present systematic review and meta-analysis evaluated the effectiveness of immersive VR distraction systems during pediatric pulp therapy procedures. Overall, the findings suggest that VR may represent an effective non-pharmacological behavioral management strategy for reducing self-reported pain and dental anxiety in children undergoing invasive dental procedures. Most included studies demonstrated favorable outcomes in subjective pain perception, anxiety reduction, behavioral cooperation, or physiological stress-related parameters. Furthermore, the quantitative synthesis demonstrated a statistically significant reduction in self-reported pain, whereas the qualitative synthesis consistently suggested beneficial effects on dental anxiety.

Behavioral and physiological findings suggest that the three stimuli most feared or most likely to generate anxiety during a dental procedure are the injection of local anesthesia, the placement of the rubber dam, and the start of tooth preparation with the handpiece. During a pulp procedure, effective control of pain and anxiety in children is essential to achieve comfort, cooperation, and compliance. A child’s cooperation can influence the decision to preserve teeth through the treatment of pulpal conditions [51].

Despite variations in study design, sample size, and outcome measures, the studies included in this review consistently indicate that VR distraction may be an effective non-pharmacological adjunct for enhancing children’s dental experiences and reducing treatment-related anxiety and pain. Overall, the findings suggest that VR serves as a valuable tool for improving behavioral and psychological outcomes in pediatric pulp therapy settings [51].

The pooled meta-analysis of self-reported pain demonstrated a significant reduction in pain scores among children treated with VR compared with conventional behavioral management techniques. Nevertheless, these findings should be interpreted cautiously because the quantitative synthesis was based on only three randomized controlled trials and showed important statistical heterogeneity (I^2^ = 93%), limiting the precision and generalizability of the pooled estimate. Accordingly, the pooled effect estimate should be interpreted cautiously and considered preliminary rather than definitive.

Regarding dental anxiety, the qualitative synthesis consistently suggested that virtual reality distraction reduced anxiety in most included studies. However, a quantitative synthesis was not performed because, after exclusion of crossover and split-mouth trials, only two parallel-group randomized controlled trials remained. These findings support the hypothesis that immersive distraction may effectively divert children’s attention away from unpleasant sensory stimuli during dental treatment. The immersive nature of VR, which combines visual and auditory isolation from the surrounding clinical environment, may explain its superior distraction potential compared with traditional audiovisual methods. By blocking external stimuli and increasing cognitive engagement, VR may reduce children’s awareness of dental instruments, clinical noises, and potentially painful procedures.

The results observed in the present are consistent with previous studies and reviews evaluating VR in pediatric healthcare and dentistry [11,16,52,53] that used VR goggles in pediatric dental treatments and observed significant reductions in pain and anxiety compared with the control group. Similarly, Goettems et al. [21] highlighted the effectiveness of non-pharmacological interventions in reducing dental anxiety and pain during pediatric dental care. Therefore, the present review expands the currently available evidence by specifically focusing on pediatric pulp therapy procedures, which are generally considered invasive and highly stressful for children.

Nevertheless, not all previous studies have demonstrated superior outcomes with VR distraction. Holmes and Girdler [54] and Koticha et al. [55] did not observe significant differences between VR goggles and conventional behavior management techniques. These discrepancies may be related to differences in the type and invasiveness of the dental procedures evaluated, since those studies mainly included children undergoing dental extractions, which may generate more intense stress and pain responses than pulp therapy procedures.

However, not all included studies demonstrated completely consistent findings across all outcome measures. Mahmoud et al. [49] reported that during rubber dam placement, administration of local anesthesia, and pulpotomy procedures, the effect of VR goggles was comparable to conventional behavioral management techniques. This discrepancy may be partially explained by age differences among participants. Younger children, particularly preschool-aged patients, tend to exhibit greater anxiety and reduced emotional regulation abilities compared with school-aged children, owing to developmental differences in cognitive maturity and coping capacity [56]. Similarly, Kasimoglu et al. [46] observed reductions in pulse rate after VR use but did not find statistically significant differences in some anxiety-related scales. These findings reinforce the multifactorial nature of dental anxiety and behavioral responses in pediatric patients. Importantly, the present findings cannot be generalized to children with special healthcare needs, who were excluded from the included studies. Future research should specifically evaluate the effectiveness of VR distraction in these patient populations.

The substantial statistical and methodological heterogeneity observed across the included studies represents an important limitation of the present review and substantially restricts the certainty and generalizability of the findings. The studies differed in design, participant age, sample size, type and invasiveness of the pulp therapy procedure, VR device and audiovisual content, duration and timing of the intervention, comparator conditions, and clinical setting. Considerable variability was also observed in the assessment methods used to evaluate pain, dental anxiety, behavioral cooperation, and physiological stress responses. Anxiety was assessed using different validated instruments, including the Modified Child Dental Anxiety Scale (MCDAS), the Venham scales, and the Facial Image Scale (FIS), whereas pain was evaluated primarily using the Wong–Baker Faces Pain Rating Scale, although one study also incorporated the FLACC behavioral pain scale. These instruments measure related but not entirely equivalent constructs, which may have contributed to the observed heterogeneity. In addition, the quantitative synthesis was based on only three parallel-group randomized controlled trials, thereby limiting the robustness and precision of the pooled effect estimate. Consequently, the magnitude of the observed benefit should be interpreted cautiously and should not be considered definitive evidence of effectiveness.

Physiological stress-related parameters also produced inconsistent findings. Two studies included in this review evaluated salivary cortisol levels as biomarkers of anxiety and stress. Mahmoud et al. [49] did not observe significant differences in salivary cortisol levels between VR and control groups, whereas Shetty et al. [47] reported significantly lower cortisol levels after treatment in children receiving VR distraction. This discrepancy may be related to differences in saliva collection protocols, timing of sample collection, or procedural characteristics. Previous studies have demonstrated that saliva collection methods may influence cortisol concentration measurements and analytical accuracy [17]. Furthermore, Chaturvedi et al. [57] and Almaummar et al. [5] reported increased salivary cortisol levels following dental extractions in children. These differences may reflect the more invasive and stressful nature of extraction procedures compared with pulp therapy.

Although the findings from subjective measures of pain and anxiety were generally favorable to VR distraction, objective physiological indicators produced less consistent results. Salivary cortisol levels were reduced in some studies but not in others, and pulse rate measurements showed variable responses. Consequently, the evidence supporting the anxiolytic effect of VR appears stronger for patient-reported outcomes than for physiological markers of stress [58].

The mechanisms underlying the beneficial effects of VR distraction are likely multifactorial. Dental anxiety in children is strongly associated with fear of dental instruments, clinical sounds, injections, and anticipation of pain. VR may reduce anxiety by completely blocking the child’s visual field and redirecting cognitive attention toward immersive audiovisual content. VR consists of several essential components, including immersion, sensory feedback, interactivity, and the creation of a computer-generated virtual environment [59,60]. These characteristics may explain its superior ability to capture children’s attention and divert cognitive focus away from unpleasant dental stimuli. Unlike traditional audiovisual distraction methods, VR integrates visual, auditory, and occasionally kinesthetic stimuli, thereby capturing a greater degree of attention and producing a stronger sense of presence within the virtual environment [61,62]. Consequently, VR may provide superior distraction compared with less immersive techniques.

Additional advantages of VR include its relative ease of use, intuitive operation, and high acceptance among both children and parents. Wismeijer and Vingerhoets [11] suggested that VR systems are generally safe and easy to administer in clinical settings without requiring extensive staff training. Hoffman et al. [63] further reported that repeated use of VR does not appear to diminish its effectiveness. Importantly, children are often willing to try VR during dental procedures, and parental acceptance of the intervention is usually high, which may facilitate its implementation in routine pediatric dental practice.

Nevertheless, the findings of this review should be interpreted with caution because several limitations were identified among the included studies. First, most studies included relatively small sample sizes, which may reduce statistical power and limit the generalizability of the findings. In addition, randomized crossover and split-mouth trials could not be included in the quantitative synthesis because the original publications did not report the paired statistical data required for appropriate meta-analysis. Consequently, the pooled estimates were based exclusively on parallel-group randomized controlled trials, reducing the amount of quantitative evidence available. Second, several studies presented methodological limitations or “some concerns” according to the RoB 2 assessment, mainly related to allocation concealment, blinding procedures, and selective reporting. Third, substantial heterogeneity was identified across studies regarding study design, VR devices, immersion levels, audiovisual content, dental procedures performed, and outcome assessment tools. This variability likely contributed to the high heterogeneity observed in the pain meta-analysis. In addition, the quantitative synthesis was based on only three parallel-group randomized controlled trials, which substantially limits the robustness of the pooled estimate despite the use of a random-effects model.

Technical limitations associated with VR use in pediatric dentistry should also be considered. Some studies described the need to adapt equipment for younger children, possible interference of the headset during clinical procedures, and limitations related to the quality of the immersive experience. Additionally, external clinical stimuli, poor audiovisual synchronization, or inadequate fitting of VR devices may reduce the effectiveness of distraction. Another important limitation is the lack of direct comparison between immersive VR systems and other distraction techniques, such as conventional audiovisual distraction or behavioral guidance strategies.

An additional consideration relates to patient-related factors that may influence the effectiveness of VR-based distraction. The included studies enrolled children across a relatively broad developmental age range, and variables such as age, cognitive maturity, baseline anxiety levels, temperament, previous dental experiences, and coping styles may affect individual responses to immersive distraction. Because of the limited number of available studies, it was not possible to explore these factors through subgroup analyses. Furthermore, all included studies were conducted in generally healthy children and excluded participants with developmental disorders, neurological conditions, or special healthcare needs. Therefore, the findings should not be generalized to the broader pediatric population. Evidence from studies evaluating audiovisual distraction strategies in children with special healthcare needs suggests that distraction-based interventions are not uniformly effective and that their success may depend on individual behavioral characteristics, communication abilities, and the manner in which the intervention is delivered. Future studies should investigate whether these patient-related factors modify the effectiveness of VR distraction during pediatric dental treatment.

Accordingly, the present conclusions apply primarily to medically healthy children undergoing pulp therapy and should not be extrapolated to children with special health care needs until specific evidence becomes available.

Despite these limitations, this systematic review presents several strengths. To the authors’ knowledge, this is the first systematic review and meta-analysis specifically evaluating the effectiveness of VR distraction during pediatric pulp therapy procedures. A comprehensive search strategy was conducted according to PRISMA guidelines, and both qualitative and quantitative syntheses were performed. Furthermore, methodological quality and certainty of evidence were assessed using internationally accepted tools, including RoB 2 and GRADE.

Although the available findings are encouraging, they should be regarded as preliminary evidence rather than as a basis for routine clinical implementation. The present results suggest that VR distraction may be considered a potentially useful adjunct to established behavioral guidance techniques, but the available evidence is insufficient to support its systematic use as a standard component of pediatric pulp therapy. Practical implementation of VR in pediatric dentistry also requires consideration of equipment cost, availability, maintenance, cleaning and infection-control procedures between patients, and possible interference of the headset with the operative field. Younger children may require additional parental support to tolerate the headset. Although discomfort or motion sickness was not specifically reported in the included studies, clinicians should remain aware of these potential practical considerations when implementing VR in routine practice. Consequently, VR should be regarded as an adjunct to, rather than a replacement for, established behavior guidance techniques. Decisions regarding its clinical use should therefore consider patient age and preferences, baseline anxiety, treatment characteristics, and the clinical setting. Further adequately powered randomized clinical trials using standardized protocols and clinically relevant outcomes are required before firm recommendations for routine practice can be made.

Future research should focus on developing larger randomized clinical trials using standardized VR protocols and outcome assessment methods. Further studies comparing different levels of immersion, audiovisual content, and distraction modalities would help identify the most effective VR strategies for pediatric dental care. Additionally, future investigations should explore the long-term behavioral effects of VR distraction and evaluate its effectiveness in different age groups, clinical settings, and dental procedures to strengthen the available evidence and optimize its implementation in pediatric dentistry.

An important priority for future research is the development of a standardized core outcome set for randomized clinical trials evaluating virtual reality distraction during pediatric pulp therapy. At a minimum, future studies should consistently assess three key domains: pain perception, dental anxiety, and behavioral cooperation. Pain should preferably be assessed using validated self-reported instruments such as the Wong–Baker Faces Pain Rating Scale (WBFPS), whereas dental anxiety should be evaluated using validated pediatric anxiety scales such as the Modified Child Dental Anxiety Scale (MCDAS) or the Facial Image Scale (FIS). Behavioral cooperation should be assessed using standardized observational instruments such as the Frankl Behavior Rating Scale. Physiological variables, including heart rate, pulse rate, or salivary cortisol, may be recorded as complementary outcomes but should not replace validated patient-reported and behavioral measures. The adoption of standardized outcomes would facilitate comparisons among studies and improve the reliability of future meta-analyses. Future trials should also pre-specify these outcomes in their study protocols and assess them at comparable clinical time points to improve consistency across studies and reduce methodological heterogeneity.

## 5. Conclusions

The findings of this systematic review and meta-analysis suggest that virtual reality distraction may represent a promising non-pharmacological adjunct for reducing self-reported pain and dental anxiety during pediatric pulp therapy. However, the available evidence is based on a small number of randomized clinical trials and is characterized by substantial statistical and methodological heterogeneity, considerable variability in outcome assessment, and low certainty of evidence. Therefore, these results should be considered preliminary and should not yet be interpreted as supporting the routine clinical implementation of VR distraction. Further adequately powered randomized clinical trials using standardized intervention protocols, comparator conditions, and validated outcome measures are required before definitive conclusions and clinical recommendations can be established.

Future randomized clinical trials should adopt standardized outcome measures for pain, anxiety, and behavioral cooperation and should specifically evaluate the effectiveness of VR distraction in children with special health care needs.

## Figures and Tables

**Table 1:** Literature search in databases.

Database	Exact Search String Used	No. of Articles	Date of Last Search
PubMed/ MEDLINE	((“Virtual Reality” [Mesh] OR “virtual reality” [Title/Abstract] OR VR [Title/Abstract] OR “virtual reality exposure therapy” [Title/Abstract] OR “immersive technolog” [Title/Abstract] OR “head mounted display” [Title/Abstract] OR “VR headset” [Title/Abstract] OR “audiovisual distraction” [Title/Abstract] OR “video eyeglasses” [Title/Abstract] OR “audio visual distraction” [Title/Abstract]) AND (“Child” [Mesh] OR “Pediatric Dentistry” [Mesh] OR child [Title/Abstract] OR pediatric [Title/Abstract] OR paediatric [Title/Abstract] OR preschool [Title/Abstract]) AND (“Pulpotomy” [Mesh] OR “Pulpectomy” [Mesh] OR pulpotom [Title/Abstract] OR pulpectom [Title/Abstract] OR “pulp therap” [Title/Abstract] OR “vital pulp therap” [Title/Abstract] OR “pulp treatment” [Title/Abstract] OR “primary molar” [Title/Abstract]) AND (dental [Title/Abstract] OR dentistry [Title/Abstract] OR “dental treatment” [Title/Abstract] OR “dental procedure” [Title/Abstract]))	21	From 2016 to 1 May 2026
Scopus	TITLE-ABS-KEY((“virtual reality” OR VR OR “virtual reality exposure therapy” OR “immersive technolog” OR “head mounted display” OR “VR headset” OR “audiovisual distraction” OR “video eyeglasses” OR “audio visual distraction”)) AND TITLE-ABS-KEY((child OR pediatric OR paediatric OR preschool OR “pediatric dentistry”)) AND TITLE-ABS-KEY((pulpotom OR pulpectom OR “pulp therap” OR “vital pulp therap” OR “pulp treatment” OR “primary molar”)) AND TITLE-ABS-KEY((dental OR dentistry OR “dental treatment” OR “dental procedure”))	23	From 2016 to 1 May 2026
EMBASE	(‘virtual reality’/exp OR ‘virtual reality’: ti, ab OR VR: ti, ab OR ‘virtual reality exposure therapy’: ti, ab OR ‘immersive technolog’: ti, ab OR ‘head mounted display’: ti, ab OR ‘VR headset’: ti, ab OR ‘audiovisual distraction’: ti, ab OR ‘video eyeglasses’: ti, ab OR ‘audio visual distraction’: ti, ab) AND (‘child’/exp OR ‘pediatric dentistry’/exp OR child: ti, ab OR pediatric: ti, ab OR paediatric: ti, ab OR preschool: ti, ab) AND (‘pulpotomy’/exp OR ‘pulpectomy’/exp OR pulpotom: ti, ab OR pulpectom: ti, ab OR ‘pulp therap’: ti, ab OR ‘vital pulp therap’: ti, ab OR ‘pulp treatment’: ti, ab OR ‘primary molar’: ti, ab) AND (dental: ti, ab OR dentistry: ti, ab OR ‘dental treatment’: ti, ab OR ‘dental procedure’: ti, ab)	15	From 2016 to 1 May 2026 March 2026

**Table 2:** Excluded studies and their reasons for exclusion.

Reasons	Excluded Studies
Studies without pulp treatment	Selvaraj et al. 2026 [29] Gardner et al. 2026 [30] Kumar et al. 2025 [31] Do et al. 2025 [32] Karuppiah et al. 2024 [33] Gala et al. 2024 [34] Prakash et al. 2025 [35] Pathak et al. 2023 [36] Ma et al. 2023 [37] Gs et al. 2021 [38]
Not using VR	Schlesinger et al. 2026 [39] Khogeer et al. 2025 [40]
Participants with any diagnosed health condition	Suresh & Shetty 2026 [41] Salama et al. 2024 [42] Mehrotra et al. 2024 [43]

**Table 3:** Characteristics of the included studies.

Authors, Year and Country	Study Design	Sample Size/Age	CG	VR Device	Pulp Treatment	Comparator	Pain Scales	Anxiety Scales	Results
Niharika et al. 2018, India [44]	RSCC	40 c/4–8 years	Yes	Google VR Box and Anti Tank VR 3D Glasses	NR	NR	WBFP	MCDA PR HR	Significant reductions in pain and anxiety scores were observed with VR use during pulp therapy.
Shetty et al. 2019, India [47]	RCT	120 c/5–8 years	Yes	i-glasses 920HR, (Ilixco Inc., Menlo Park, CA, USA)	Pulpotomy	Conventional distraction TSD voice control	WBFP	MCDA Salivary cortisol	VR significantly reduced pain and anxiety. Salivary cortisol levels decreased significantly in VR group.
Alshatrat et al. 2025, Jordan [45]	RCT	154 c/ 5–12 years	Yes	iWear (Vuzix^®^, Rochester, New York, NY, USA)	NR	NR	WBFP FLACC VA	NR	VR resulted in a significant reduction in pain perception and doubled the level of relaxation.
El Haleem et al. 2022, Egypt [48]	RCT	60 c/ 4–6 years	Yes	NR	Pulpotomy	Non-pharmacological management	WBFP	VP FBR	VR significantly improved behavior and reduced anxiety and pain.
Mahmoud et al. 2022, Egypt [49]	RCT	20 c/ 4–5 years	Yes	NR	Pulpotomy	TSD, conventional distraction positive reinforcement	NR	VCAR Salivary cortisol	VR showed significantly lower VCARS scores. No significant differences in salivary cortisol levels were observed.
Bahrololoomi et al. 2024, Iran [50]	SRCC	30 c/ 6–8 years	Yes	LEJI VR Mini glasses, China	Pulpotomy	NR	WBFP	MCDA PR	PR wasnt significantly different. VR showed significantly lower MCDAS and WBFPS scores.
Kasimoglu et al. 2025, Turkey [46]	3-arm parallel-group RCT	90 c/ 6–10 years	Yes	NR	Pulpotomy	Cartoon TSD positive reinforcement	PR	FI CFSSD FBR PR	PR significantly decreased after treatment. No significant differences were in FIS, CFSSD, or FBRS.

RSCC: randomized single-blind crossover clinical trial; RCT: randomized controlled trial; SRCC: Split-mouth randomized crossover clinical trial; c: children; CG: control group; VR: virtual reality; TSD: tell-show-do; WBFP: Wong–Baker Faces Pain; FLACC: Face, Legs, Activity, Cry, Consolability; VA: Visual Analog; FI: Facial Image; MCDA: Modified Child Dental Anxiety; CFSSD: Children’s Fear Survey Schedule Dental; VCAR: Venham Clinical Anxiety Rating; VP: Venham Picture; FBR: Frankl Behavior Rating; PR: pulse rate; HR: heart rate; NR: not reported.

**Table 4:** Mean pain scores and standard deviations of VR and control groups included in the self-reported pain meta-analysis.

Study	Scale	VR *n*	VR Mean	VR SD	Control *n*	Control Mean	Control SD
Shetty et al. 2019 [47]	WBFPS	58	2.42	1.47	60	5.60	1.22
Alshatrat et al. 2025 [45]	WBFPS	17	3.41	3.14	14	6.71	3.81
El Haleem et al. 2022 [48]	WBFPS	30	5.00	1.88	30	6.27	2.72

**Table 5:** Risk of bias assessment.

Study	Randomization Process	Deviations from Intended Interventions	Missing Outcome Data	Measurement of Outcome	Selection of Reported Result	Overall RoB
Niharika et al. 2018 [44]	Some concerns	Some concerns	Low risk	Low risk	Some concerns	Some concerns
Shetty et al. 2019 [47]	Some concerns	Some concerns	Low risk	Low risk	Some concerns	Some concerns
Alshatrat et al. 2025 [45]	Low risk	Low risk	Low risk	Low risk	Some concerns	Low risk
El Haleem et al. 2022 [48]	Some concerns	Some concerns	Low risk	Low risk	Some concerns	Some concerns
Mahmoud et al. 2022 [49]	Some concerns	Some concerns	Low risk	Low risk	Some concerns	Some concerns
Bahrololoomi et al. 2024 [50]	Some concerns	Some concerns	Low risk	Low risk	Some concerns	Some concerns
Kasimoglu et al. 2025 [46]	Some concerns	Some concerns	Some concerns	Low risk	Some concerns	Some concerns

**Table 6:** Summary of Findings (GRADE).

Outcome	No. of Studies	Study Design	Risk of Bias	Inconsistency	Indirectness	Imprecision	Publication Bias	Certainty of Evidence
Self-reported pain (meta-analysis)	3	RCTs	Serious	Serious	Not serious	Not serious	Undetected	⨁⨁◯◯ Low
Dental anxiety (qualitative synthesis)	4	RCTs	Serious	Serious	Not serious	Not serious	Undetected	⨁⨁◯◯ Low
Physiological parameters (heart rate, pulse, cortisol, etc.)	4	RCTs	Serious	Serious	Serious	Very serious	Undetected	⨁◯◯◯ Very low

## Data Availability

No new data were created or analyzed in this study. Data sharing is not applicable to this article.

## Supplementary Materials

The following supporting information can be downloaded at: https://www.mdpi.com/article/10.3390/dj14080517/s1, Table S1: PRISMA 2020 Checklist [23].
